# Individual alpha frequency tACS reduces static functional connectivity across the default mode network

**DOI:** 10.3389/fnhum.2025.1534321

**Published:** 2025-05-14

**Authors:** Martín Carrasco-Gómez, Alejandra García-Colomo, Jesús Cabrera-Álvarez, Alberto del Cerro-León, Carlos J. Gómez-Ariza, Andrés Santos, Fernando Maestú

**Affiliations:** ^1^Department of Electronical Engineering, E.T.S. de Ingenieros de Telecomunicación, Universidad Politécnica de Madrid, Madrid, Spain; ^2^Center for Cognitive and Computational Neuroscience, Complutense University of Madrid, Madrid, Spain; ^3^CIBER de Bioingeniería, Biomateriales y Nanomedicina (CIBER-BBN), Instituto de Salud Carlos III, Madrid, Spain; ^4^Department of Experimental Psychology, Cognitive Psychology and Speech and Language Therapy, Complutense University of Madrid, Madrid, Spain; ^5^Department of Psychology, University of Jaén, Jaén, Spain; ^6^Health Research Institute of the Hospital Clínico San Carlos (IdISSC), Madrid, Spain

**Keywords:** tACS, functional connectivity, magnetoencephalography, cortical folding, individual alpha frequency

## Abstract

**Introduction:**

Research on the influence of transcranial alternating current stimulation over alpha functional connectivity (FC) is scarce, even when it poses as a potential treatment for various diseases. This study aimed to investigate the effects of individual alpha frequency tACS (IAF-tACS) on FC within the default mode network (DMN) in healthy individuals, particularly following the triple network model.

**Materials and methods:**

27 healthy participants were recruited, who underwent a 20-min IAF-tACS session over parieto-occipital areas and three magnetoencephalography (MEG) recordings: two pre-stimulation and one post-stimulation. Participants were randomly assigned to either the stimulation or sham group. Both dynamic FC (dFC) and static FC (sFC) were evaluated through the leakage corrected amplitude envelope correlation (AEC-c). Statistical analyses compared both Pre-Post FC ratio between groups through ratio *t*-tests and intragroup FC changes through repeated measures *t*-tests, with FDR correction applied to account for multiple comparisons. An additional analysis simulated the influence of the cortical folding on the effect of tACS over FC.

**Results:**

IAF-tACS significantly decreased sFC in intra- and inter-DMN links in the stimulation group compared to the sham group, with a special influence over antero-posterior links between hubs of the DMN. Negative correlations were found between AEC-c sFC changes and power alterations in posterior DMN areas, suggesting a complex interaction between cortical folding and electric field direction. On the other hand, dFC increased in both sham and stimulation groups, and no between-group differences were found.

**Conclusion:**

Against our initial hypothesis, IAF-tACS reduced sFC in the DMN, possibly due to phase disparities introduced by cortical gyrification. These findings suggest that tACS might modulate FC in a more complex manner than previously thought, highlighting the need for further research into the personalized application of neuromodulation techniques, as well as its potential therapeutic implications for conditions like Alzheimer’s disease.

## 1 Introduction

The brain shows spatio-temporally organized patterns of activity even when at wakeful rest. During this resting state, the alpha rhythm (8–12 Hz) becomes the predominant oscillation over posterior regions (Buzsáki, 2006). The spontaneous emergence of alpha in resting state, and its correlation with performance, has led to the hypothesis that alpha reflects a functional inhibitory role that is key to the allocation of processing resources for environmental stimuli (Jensen and Mazaheri, 2010). In addition to the alpha rhythm, resting state is associated with changes in functional connectivity (FC) understood as statistical relationships between brain signals over time (Friston, 1994, 2011). These functional interactions give rise to brain networks that reflect interregional communication to support cognitive function, forming the so-called intrinsic connectivity networks, which represent consistent patterns of strong coupling of the ongoing activity. From the several stable ICNs identified in the human brain, three are of particular importance for the understanding of cognitive function and dysfunction composing what Menon, 2011 proposed to call the triple network model. Within the triple model network, the default mode network (DMN) is a key functional network that is active during resting periods and deactivates during task performance (Raichle, 2011). The DMN is involved in memory processing (Menon, 2011) and includes regions such as the medial prefrontal cortex, middle temporal gyrus, the hippocampus, the posterior cingulate cortex (PCC) and the precuneus (PCU), the latter standing out as a hub of the network (Alves et al., 2019; Utevsky et al., 2014). Disruptions in both the alpha rhythms and the activity in the DMN have been associated with multiple brain diseases, such as Alzheimer’s disease or depression (Babiloni et al., 2009; Canuet et al., 2015; López-Sanz et al., 2016), therefore, their modulation constitutes a potential therapeutic avenue to explore. Completing the triple model network, the other two networks include the central executive network (CEN), involved in externally oriented attention, and the salience network (SN), mediating between the DMN and CEN. These two networks show increased activity during stimulus-driven cognitive and affective information processing, and are affected by other neurological disorders (Menon, 2011).

Transcranial alternating current stimulation (tACS) is a non-invasive stimulation technique capable of modulating oscillatory activity in the brain and inducing neuroplasticity (Chaieb et al., 2011). Weak alternating electrical currents are applied to the scalp, reaching the brain, influencing neurons’ membrane potentials and thus, altering neuronal probability of activation (Elyamany et al., 2021). As a result, the application of tACS can induce neural entrainment (i.e., the synchronization of an oscillatory system to an external driver) at the frequency of stimulation (Liu et al., 2018). The anatomical and functional complexity of the brain makes the results of neuromodulation highly dependent on the protocol employed (Antal et al., 2008), but it has been observed that entrainment is more likely to occur if the stimulation frequency matches the frequency of the ongoing oscillation (Vogeti et al., 2022) and if the anatomy of the target region is taken into account (Aberra et al., 2020; Cabrera-Álvarez et al., 2023).

Externally applied oscillatory currents can interact with the endogenous alpha rhythm to enhance or disrupt it (De Koninck et al., 2023; Liu et al., 2018). In this line, tACS at the individual alpha frequency (IAF) has been shown to increase the power of alpha rhythm after stimulation with effects remaining up to 70 min after the protocol (Kasten et al., 2016; Zaehle et al., 2010). These after-effects likely involve plastic changes in neuronal circuits (Vossen et al., 2015).

Beyond these effects, cortical modulation may also influence FC. While, to the best of our knowledge, no studies have investigated the effect of tACS on dynamic FC (dFC), recent research suggests that tACS modulates static FC (sFC) in a frequency- and network-specific manner, influencing connectivity over the networks related to the ongoing activity, and increasing FC around tACS stimulation frequency (Antal and Paulus, 2013). For instance, occipito-parietal alpha stimulation during resting-state increases sFC specifically in the alpha band and in the regions of the DMN (Clancy et al., 2018; Schwab et al., 2019), which were suggested in the latter to be mediated by changes in alpha power induced by stimulation (Clancy et al., 2018). In contrast, a 6 Hz stimulation in resting state–when the brain is typically oscillating in alpha–decreases sFC in the DMN (Abellaneda-Pérez et al., 2020), highlighting the variability in tACS effects depending on the stimulation frequency. These modulations of sFC have been shown to enhance cognitive performance when increasing interregional communication through coherence (Helfrich et al., 2014; Kar et al., 2020; Polanía et al., 2012; Preisig et al., 2021; Tan et al., 2019). Therefore, tACS has become a promising tool for modulating FC both in health and disease, and could help in understanding the nature of both sFC and dFC in an interventionist approach, rather than just observational.

In this study, we aimed to investigate the influence of a personalized IAF-tACS protocol on healthy individuals’ dFC and sFC through MEG recordings. According to the aforementioned studies, we expected to find an increased sFC in the participants’ DMN after the stimulation, while no direction was assumed for the changes in dFC due to the lack of literature in the matter. To get a spatially finer analysis and assess whether the influence of tACS is restricted to the DMN alone, we evaluated FC alterations due to tACS under the triple network model (Menon, 2011). We hypothesized that tACS would have a greater effect over the DMN for two reasons: (i) the electric field elicited by parieto-occipital tACS was stronger in areas belonging to the DMN, and (ii) the DMN is the most active network under resting conditions. Finally, we relate changes in FC to changes in power in the areas of interest.

## 2 Materials and methods

### 2.1 Study design

The study comprised one tACS session and three magnetoencephalography (MEG) scans, two before (Pre1 and Pre2 sessions) and one after the stimulation (Post session). We firstly recorded the electrophysiological activity of each participant through two successive 5-min eyes-closed resting-state MEG recordings, with a 10-min interval between sessions. Two MEG recordings before the stimulation were included to account for any possible variability in the individual alpha-peak frequency (IAF) of the participants. The IAF was derived from each recording using a fast-preprocessing algorithm described in the “MEG preprocessing and source reconstruction” section, and then averaged. Statistical analyses for the current paper were carried out using the Pre2 data as the baseline measure since it was closer in time to the stimulation and all participants had experienced a similar situation until that point. Participants were then randomly assigned to either the real stimulation (stim) or placebo (sham) group, and received 20 min of stimulation at their own IAF with their eyes closed. Immediately after the stimulation, we performed a third MEG recording to measure its effects on the participants. We decided to use a resting-state eyes-closed paradigm given its prominent alpha activity, and previous work on the same kind of stimulation (Wang et al., 2022; Zarubin et al., 2020).

### 2.2 Participants

We recruited 27 (11 female) participants aged between 22 and 55 years from the Center for Cognitive and Computational Neuroscience (C3N) at the Complutense University of Madrid (UCM). The study included only right-handed, native Spanish-speaking participants without any prior neuropsychiatric history or metallic prostheses that could interfere with neuroimaging and neuromodulation. This population was selected because we were first interested in obtaining baseline tACS effects against which to compare those obtained in subsequent studies with clinical populations. Additionally, participants with indistinguishable IAF, i.e., no noticeable prominent peak in the spectra plot, were excluded. We adhered to current guidelines and safety regulations throughout the research and obtained informed consent from each participant before their involvement.

### 2.3 MEG data acquisition

MEG signals were acquired during 5 min of eyes-closed resting state at 1 kHz sampling rate, using 306 channels (102 magnetometers and 204 gradiometers) whole-head Elekta Neuromag system (Elekta AB, Stockholm, Sweden) located in a magnetically isolated room (VacuumSchmelze GmbH, Hanau, Germany). Using a Fastrak 3D digitizer (Polhemus, Colchester, Vermont), the positions of four head position indicator (HPI) coils attached to the scalp were defined and the shape of each participant’s head relative to three anatomical locations (nasion and both preauricular points) was modeled. An online anti-aliasing filter [0.1–330 Hz] was applied during the whole session.

### 2.4 MEG preprocessing and source reconstruction

The Maxfilter software (v.2.2, correlation threshold = 0.9, time window = 10 s) was used to remove the environmental noise in the raw data, using the temporal extension of the signal space separation (tSSS) method with movement compensation (Taulu and Simola, 2006). For further analysis, only data from all magnetometers was considered, given the redundancy in gradiometer data after tSSS (Garcés et al., 2017). Physiological and jump artifacts were located using the automatic Fieldtrip software (Oostenveld et al., 2011) and later reviewed by MEG signal experts. Lastly, the clean signal was divided into 4 s-segments and eye-blinks and cardiac artifacts were removed with an independent component analysis (ICA) based on SOBI (Belouchrani et al., 1997). All segments with physiological artifacts were discarded. Preprocessed MEG data was then used to carry out source localization using a linearly constrained minimum variance (LCMV) beamformer (Van Veen et al., 1997). Since not all subjects had available T1 MRIs, a 1 mm resolution template of healthy adults normalized to the Montreal Neurological Institute (MNI) with a 1 mm voxel size template was used to place the sources inside the brain in a homogeneous grid of 1 cm. Next, both the template and the grid were linearly transformed to fit the head shape of each subject and a local spheres approach was used to define the leadfields and fit the headshape of each subject in the vicinity of each sensor. Using the computed leadfield and the average of all the covariance matrices for each segment, we computed the spatial filter coefficients, which were used to estimate the source-space time-series for each source in all segments. A total of 1,210 source positions belonging to 80 regions of interest defined by the automated anatomical labeling atlas (Tzourio-Mazoyer et al., 2002) were identified. The remaining sources are not part of cortical regions defined by the atlas (i.e., white matter, CSF, or subcortical regions) and thus are not considered as source generators of MEG signal (Hämäläinen et al., 1993).

To determine the IAF we used a fast-processing algorithm immediately after the acquisition of the pre1 and Pre2 recordings. Some steps of the aforementioned preprocessing pipeline were omitted to obtain the IAF for the following stimulation in said frequency. Specifically, no tSSS or movement compensation method was applied, and manual revision of artifacts was skipped as well. ICA was used to remove physiological artifacts and contaminated segments were manually removed. The power spectrum for each magnetometer was calculated using DPSS, and then averaged. The resulting spectra from occipito-parietal channels were visually inspected, and the frequency of the power peak in the alpha band (8–12 Hz) was defined as the IAF. The IAFs from sessions Pre1 and Pre2 were averaged to determine the final frequency for the neuromodulation procedure.

### 2.5 Neuromodulation

**FIGURE 1:**
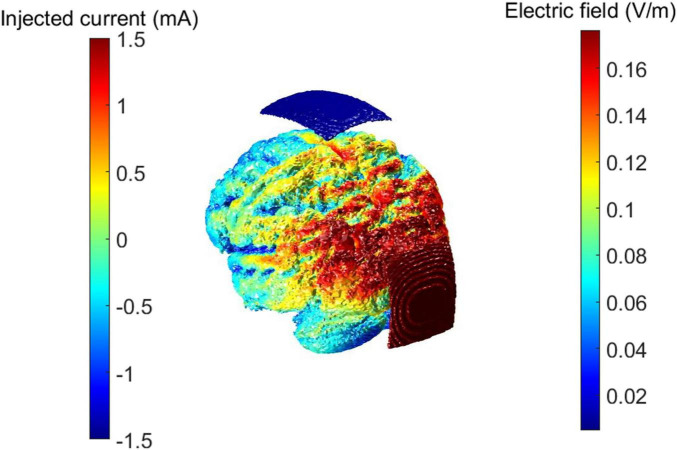
tACS maximal electric field distribution and example of the setting in a patient. Electric field distribution was estimated with ROAST, a software toolbox for MATLAB focused on simulation of electric fields on brain volumes through transcranial electrical stimulation. Red and blue patches represent the sponge electrodes positions over the participant’s scalp.

The neuromodulation sessions utilized bipolar tACS with two conductive rubber electrodes (7 cm × 5 cm) positioned at Cz (midline central) and Oz (midline occipital), and followed the same protocol as previous studies that obtained significant effects over the alpha power (Kasten et al., 2016; Zarubin et al., 2020) focusing on occipitoparietal areas where relevant hubs of the DMN network (i.e., precuneus) are located (Alves et al., 2019; Utevsky et al., 2014). A microprocessor-controlled device, the NeuroConn DC-StimulatorPlus (Neurocare, Ilmenau, Germany) was used for delivering alternating current. The electrodes were covered with sponges soaked in saline solution. For the stim group, stimulation at the IAF was applied during a 20-min session with a current intensity of 3 mA peak-to-peak. In contrast, participants in the sham group were only exposed to stimulation during the fade-in and fade-out periods, each lasting 30 s. Figure 1 shows the maximum elicited electric field as simulated with ROAST (Huang et al., 2019) and an example of the setting in a participant. After completing the stimulation and recording protocols, participants responded to a questionnaire on potential adverse effects of tACS (Brunoni et al., 2011).

### 2.6 Functional connectivity

**FIGURE 2:**
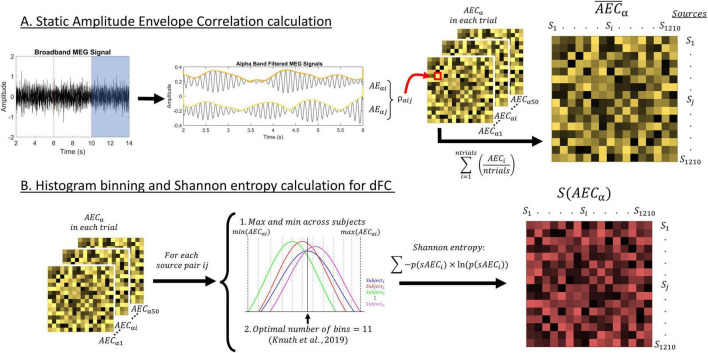
Depiction of the methods used in this study. **(A)** Description of the procedure to calculate the static AEC-c, including the signal filtering, envelope extraction through the Hilbert transform, and correlation between envelopes. **(B)** Description of the procedure to calculate the entropy for each source pair timecourse. We used a histogram with 11 bins to calculate Shannon entropy. This resulted in a value of entropy for each source pair and participant S(AEC), representing their dFC.

FC was estimated after zero-phase filtering the data in the IAF ± 2 Hz frequency band through a finite impulse response filter with an order of 1,800, designed with a Hamming window. Figure 2 depicts the calculation process of FC parameters in this study.

#### 2.6.1 Static functional connectivity

Amplitude-derived connectivity was calculated through the amplitude envelope correlation with leakage correction [AEC-c (O’Neill et al., 2015)] between the time series of each pair of the 1,210 cortical sources. After orthogonalization, making it insensitive to volume conduction, the absolute value of the Pearson correlation between the time series’ envelopes was used. AEC-c was chosen because of its reliability, and within and between subject consistency (Colclough et al., 2016). This sFC parameter ranges from 0 to 1, that is, no connectivity to full connectivity.

We calculated AEC-c in each of the defined signal segments, and then averaged over those, obtaining the so-called static FC (sFC), resulting in connectivity matrices of dimension 1,210 sources × 1,210 sources per participant. These matrices were then reduced to 40 ROIs × 40 ROIs by grouping sources and averaging their FC according to their position in the automated anatomical labeling atlas (Tzourio-Mazoyer et al., 2002), resulting in 1,210 cortical sources distributed across 40 regions of interest (80 unilateral regions grouped in bilateral analogs).

#### 2.6.2 Dynamic functional connectivity

We assessed dynamic FC (dFC) through the entropy of the FC timecourses of AEC-c, following the same methodology applied in Carrasco-Gómez et al. (2024). Starting from the unaveraged AEC-c connectivity matrices, of dimensions 1,210 sources × 1,210 sources × 50 segments, the entropy of each of those FC time series was calculated by means of histograms with linearly spaced bins.

Firstly, we determined the number of bins for the creation of optimal histograms in our dataset, following the guidelines exposed by Knuth (2019) for each of the pairwise source connectivity terms time series in all our participants. We decided to use the median of the resulting calculations, and obtained an optimal number of 11 bins. Then, and to ensure comparability between the subjects’ dFC matrices, we defined the minimum and maximum values for the binning procedure as the minimum and maximum value of the AEC-c at each of the pairwise connectivity terms across all participants. Finally, following the optimal binning procedure, the Shannon entropy was calculated (Shannon, 1948):


$$S\,\left(A\,E\,C\right)=-\sum_{n\,b\,i\,n\,s}^{i=1}p\,\left(A\,E\,C_{i}\right)\times L\,n\,\left(p\,\left(A\,E\,C_{i}\right)\right)$$

Where S is the entropy operator, *AEC* is our time series of connectivity strengths, p(*AEC*_i_) is the probability for connectivity strength to fall under bin i, *Ln* is the natural logarithm and nbins is the total number of bins. The resulting entropy matrices were symmetric and included one value of entropy for each of the 1,210 sources × 1,210 sources pairwise connectivity terms. Each of these values represents the level of dynamicity of the connectivity term over time. In other words, each connectivity term was assigned a value which portrays how variant the FC was over time, with higher values indicating a higher variability and vice versa. Finally, and equally as for sFC parameters, the connectivity matrices were reduced to matrices of dimensions 40 ROIs × 40 ROIs.

### 2.7 Statistical analyses

Initially, demographic characteristics were evaluated, using an independent sample *t*-test to compare age and IAF, as well as a chi-square test to compare sex proportion between each of the groups. We also performed a baseline (Pre2) comparison of FC at the DMN between the *stim* and *sham* groups.

To evaluate the influence of the stimulation protocol on sFC and dFC, we performed two types of *t*-tests: (1) independent samples *t*-tests to compare the FC changes in *stim* versus *sham* groups, using a measure of their FC ratio of change; (2) repeated measures *t*-test to evaluate the change in FC between the Pre2 and Post per experimental group.

For the former, FC ratio of change was calculated by dividing the FC of the *Post* session of each subject by that of the *Pre2* session. Next, we calculated the logarithm of the ratio to eliminate the statistical asymmetry of ratios and obtain statistical distributions centered in 0, where negative values represent decreases and positive values represent increases over recording sessions:


$$F\,C_{r\,a\,t\,i\,o}=l\,o\,g\,\left(\frac{F\,C_{P\,o\,s\,t}}{F\,C_{P\,r\,e\,2}}\right)$$

As a clarification, all analyses were performed comparing *stim* vs. *sham* groups, and *Post* vs. *Pre2* stages, so that a positive Cohen’s d or t-statistic would represent increased FC values in the *stim* group or *Post* stage.

Firstly, we evaluated the FC between the three networks in the model proposed by Menon (2011): DMN, SN and CEN. To do so, we calculated the aforementioned statistical comparisons for each of the networks intraconnectivity (DMN-DMN, SN-SN, CEN-CEN), and the pairwise connectivity between each of the networks (DMN-SN, DMN-CEN, SN-CEN). The set of tests were FDR corrected with a *q* = 0.1 to compensate for the multiple comparisons (Benjamini and Hochberg, 1995). The regions included in each of these networks can be found in Supplementary Table 1.

Secondly, to evaluate which areas of the DMN drive the differences in connectivity change between the *stim* and *sham* groups, we conducted post-hoc *t*-tests comparing the FC ratio at each link between any two areas belonging to the DMN. Given the high number of comparisons, *post hoc* results were FDR corrected with a q of 0.1 as well.

Lastly, we aimed to study the relationship between the FC ratios and simultaneous alterations in relative power given the previously suggested capacity of tACS to alter power (Cabrera-Álvarez et al., 2023). To do this, we performed a Pearson’s correlation between the FC ratio of those DMN links that showed significant differences in the *stim-sham* comparison after FDR correction, and the ratio of IAF ± 2 Hz relative power of the regions that make up the link (e.g., supposing the ACC-PCC link’s ratio showed significant differences between groups, the FC ratio would be correlated with the power ratio of both the ACC and PCC), independently. This analysis was carried out separately for the *stim* and *sham* groups, and their rhos were compared through a z-test on Fisher z-transformed correlation coefficients (Hinkle et al., 1988).

All calculations were performed in MATLAB 2021b.

### 2.8 Simulation of tACS phase-shifts effects on FC

We performed an *ad-hoc* theoretical analysis to investigate the potential effects of cortical morphology over FC modulation. Given our current stimulation protocol and cortical folding, the neurons of certain regions may receive the stimulation in anti-phase fashion, as the electric field direction may result anti-parallel to some cortical columns (i.e., hyperpolarizing) and parallel to others (Cabrera-Álvarez et al., 2023), as shown in Figure 6. To evaluate the effect of these phase disparities over sFC, we simulated two MEG signals through filtered white noise and calculated their initial connectivity through AEC. Afterward, we added a sinusoidal stimulus to each of them with different phase shifts and calculated again AEC. This procedure was repeated 100 times.

A repeated measures *t*-test was performed to compare the connectivity values between the cases of no-stimulation, sinusoid stimulation with 0 phase shift, and sinusoid stimulation with π phase shift. Supplementary Material 1 explains the complete methodology for the simulation process.

## 3 Results

### 3.1 Sample demographics and adverse tACS effects

Sample demographics and variables of interest used in this study are shown in Table 1, and baseline FC and reported tACS effects are depicted in Supplementary Table 2. No statistically significant differences were observed between the groups either in demographics or tACS adverse aftereffects, or in baseline FC.

**TABLE 1:** Sample demographics.

	*Stim*	*Sham*	Stat	*p*-value
Age	33.4 ± 8.4	32.2 ± 9.0	0.3940	0.7302
Sex	9M/5F	6M/6F	0.5403	0.4623
IAF	10.3 ± 0.9	10.5 ± 1.3	−0.5741	0.5713

The differences in each of the demographic variables are shown in the two rightmost columns, displaying the t-statistic or chi-square statistic for continuous and categorical variables, respectively, and *p*-value. IAF, individual alpha frequency.

### 3.2 DMN’s sFC decreases through tACS

**FIGURE 3:**
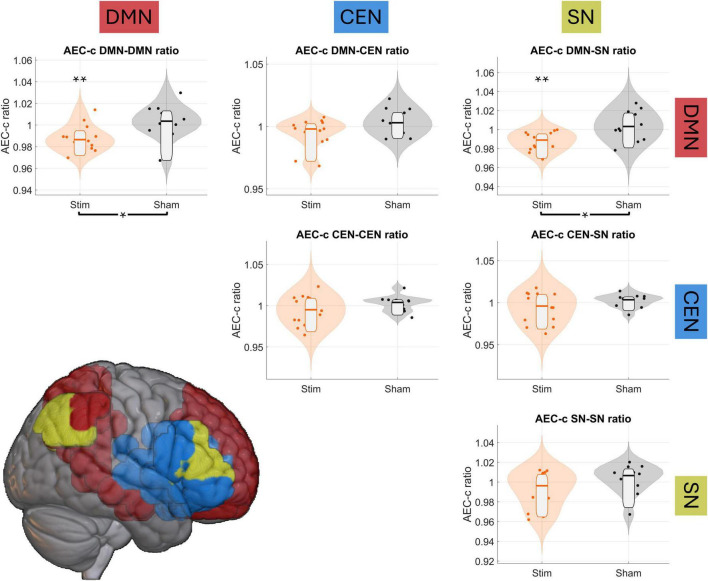
tACS reduces DMN’s connectivity. Violin plots show the *Post*/*Pre2* FC ratio among the triple network, with orange violin plots corresponding to the *stim* group and the gray ones to the *sham* group. Single asterisk (*) and brackets below the graphs indicate significant between-group differences in the FC ratio, while double asterisks (**) above each violin plot indicate significant intra-group pre/post differences. The brain render at the bottom of the figure displays the networks included in this analysis: default mode network (DMN) in red, central executive network (CEN) in blue, and salience network (SN) in yellow.

To assess the effect of tACS on the DMN’s FC, and evaluate whether this effect was exclusive to this network, we performed independent samples ratio *t*-tests comparing FC change between the *stim* and *sham* groups guided by the triple network model (Menon, 2011). Then, we conducted repeated measures *t*-tests to evaluate statistically the change from *Pre2* to *Post* stimulation for each group. FC was thus evaluated in six different relationships: DMN-DMN, DMN-SN, DMN-CEN, CEN-CEN, CEN-SN and SN-SN. FDR correction (*q* = 0.1, *n*_*comparisons*_ = 36) yielded a critical *p*-value of 0.0086. Figure 3 shows the results for these analyses.

Regarding sFC changes, independent samples *t*-tests found significant decreases of sFC between *sham* and *stim* groups for the DMN-DMN and DMN-SN spatial relationship, as shown in Figure 3: DMN-DMN [t-stat = −2.8614, d-Cohen = −1.1257, *p*-value = 0.0086], DMN-SN [t-stat = −3.3155, d-Cohen = −1.3043, *p*-value = 0.0029]. Decreases of sFC between DMN-CEN, while initially significant, did not survive FDR correction [t-stat = −2.0967, d-Cohen = −0.8249, *p*-value = 0.0467]. Repeated-measures *t*-test showed significant sFC reductions between *Pre2* and *Post* sessions in the *stim* group: DMN-DMN [t-stat = −3.9109, d-Cohen = −0.6383, *p*-value = 0.0018], DMN-SN [*t*-test = −4.7026, d-Cohen = −0.6507, *p*-value = 0.0004]. The rest of the tests were non-significant, and a complete report on all statistical tests can be found in Supplementary Table 3.

While independent samples ratio *t*-tests did not reveal between-group differences in dFC in any of the intra- or internetwork comparisons, repeated-measures *t*-tests found significant pre-post increases in both sham and stim groups. The sham group only showed a significant increase in the intra DMN link (DMN-DMN: [t-stat = 3.2022, d-Cohen = 0.6615, *p*-value = 0.0084]), whereas the stim group showed significant intra- and inter-DMN increases (DMN-DMN: [t-stat = 3.4317, d-Cohen = 0.4755, *p*-value = 0.0045]; DMN-CEN: [t-stat = 3.8618, d-Cohen = 0.4771, *p*-value = 0.0020]; DMN-SN: [t-stat = 3.5583, d-Cohen = 0.4825, *p*-value = 0.0035]). The remaining comparisons were nonsignificant or did not survive FDR correction, as shown in Supplementary Table 3. Since no between-group differences were detected for dFC, no post-hoc analyses were performed for this measure in the next sections.

### 3.3 tACS prominently decreases sFC between posterior-posterior and antero-posterior hub links of the DMN

Additional *post hoc* statistical tests were performed to identify the links between the DMN areas, defined as including the following AAL regions (Raichle, 2015): superior frontal gyrus (SFG), gyrus rectus (Rectus), hippocampus (Hip), anterior cingulate cortex (ACC), posterior cingulate cortex (PCC), inferior parietal gyrus (IPG), and the precuneus (PCU) driving the between-group results presented in the previous section. Given the high number of comparisons (*n*_*comparisons*_ = 28), results were FDR-corrected with a q = 0.1, which yielded a critical *p*-value of 0.0244.

**FIGURE 4:**
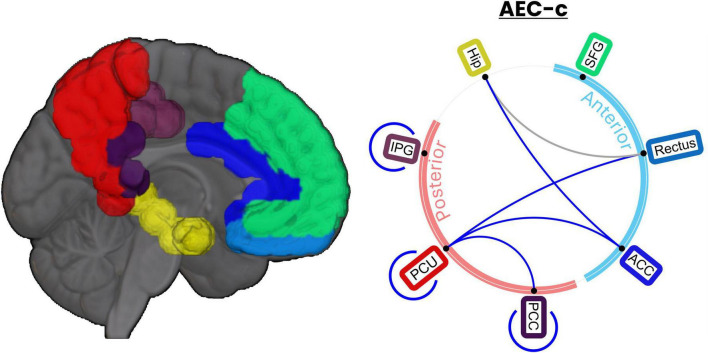
DMN links show a significant decrease in connectivity after tACS. Ratio *t*-tests revealed 8 DMN links showing a significant AEC-c reduction when comparing *stim* and *sham* groups. Of those, 7 AEC-c links still showed significance after FDR correction (*q* = 0.1). Links significant after FDR correction are indicated in blue, while those that lost significance are indicated in gray.

Out of the 28 possible links, 7 showed statistically significant differences after FDR correction using AEC-c including Hip-ACC, IPG-IPG, PCC-PCC, PCU-Rectus, PCU-PCC, PCU-ACC, and PCU-PCU, as shown in Figure 4. These differences were in the same direction as reported previously (i.e., a decrease in the FC of the stim group). Supplementary Table 4 includes a complete statistical report for the 28 possible links.

### 3.4 tACS-induced sFC change of posterior areas correlates with power change

To understand the relationship between sFC and power changes elicited by the stimulation, we performed a *post hoc* analysis correlating the AEC-c value of the significant links reported in the previous section and the relative power of the IAF ± 2 Hz of each area involved. This represented 11 Pearson’s correlations per group, as self-connections (e.g., PCU-PCU) involved one test, while inter-regional connections (e.g., PCU-PCC) involved two.

**FIGURE 5:**
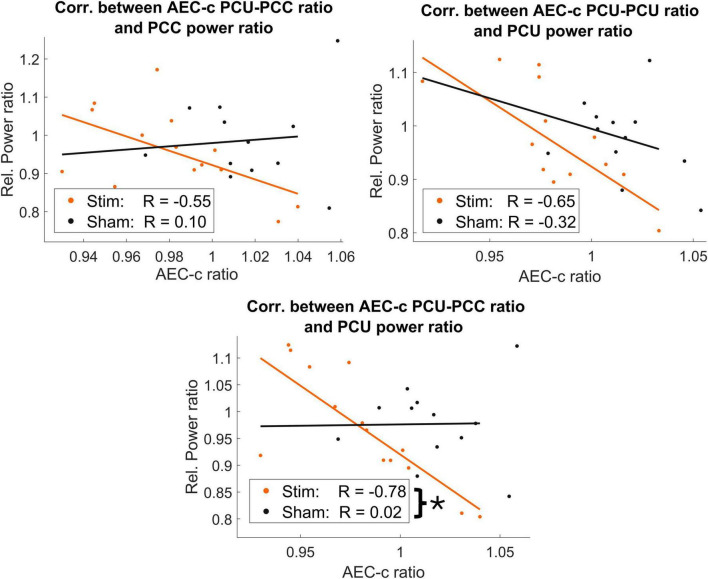
Negative correlations between AEC-c FC ratio in posterior areas and IAF ± 2 Hz relative power. Significant correlations between AEC-c and relative power in the *stim* group (orange) were found in posterior regions, while these correlations were nonsignificant in the *sham* group (black). However, only the correlation between the PCC-PCU and the IAF ± 2 Hz relative power at the PCU was able to significantly differentiate between the *stim* and *sham* groups, as indicated by a single asterisk (*).

While no significant correlations were found in the sham group, significant negative correlations between FC and normalized power in the stim group were obtained in two out of the seven links studied: PCU-PCU and PCU-PCC correlating with both regions relative power (Figure 5). Nevertheless, the only case that exhibited a significantly different correlation pattern between stim and sham groups was that between the PCC-PCU’s sFC and the normalized power of the PCU [z-stat = −2.3703, *p*-value = 0.0178]. Importantly, no differences in the relative power in the IAF ± 2 Hz band were found between the stim and sham groups in any of the studied regions.

### 3.5 Simulating tACS-induced phase disparities explain the reductions of FC

**FIGURE 6:**
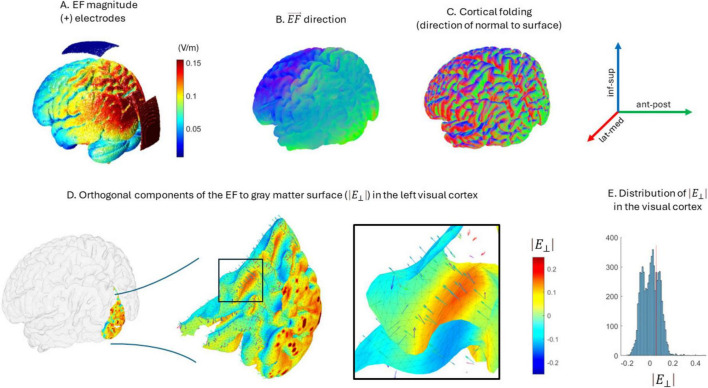
Orthogonal components of the electric field with respect to the white-matter surface. **(A)** Electrode location and electric field magnitude over the brain. **(B,C)** show the direction of both the electric field given the protocol **(B)** and the direction of the normal component to white matter surface in the folded cortex **(C)**. **(D)** Distribution of orthogonal components of the electric field in the visual cortex. A sample of vectors is shown indicating both the direction of the electric field and the normal component to the surface. The surface color indicates the magnitude of the orthogonal component (red-depolarizing, blue-hyperpolarizing). **(E)** Distribution of magnitudes of the orthogonal components of the electric field for the visual cortex. Note that in the same region coexist positive and negative values, and therefore, anti-phase stimulation effects.

As stated in the introduction, while we expected to observe an increase in sFC after the stimulation protocol, the results showed a generalized decrease in sFC in the *stim* group. To understand further these results, we studied the potential interaction of cortical morphology with the stimulation protocol. Given the brain cortical folding, the neurons of certain regions may receive the stimulation in anti-phase, as the electric field direction may result antiparallel to some cortical columns (i.e., hyper-polarizing effect) and parallel to others (Cabrera-Álvarez et al., 2023; see Figure 6). Therefore, we performed a theoretical experiment by introducing phase disparities in two simulated MEG signals and measuring the resulting sFC through AEC.

**FIGURE 7:**
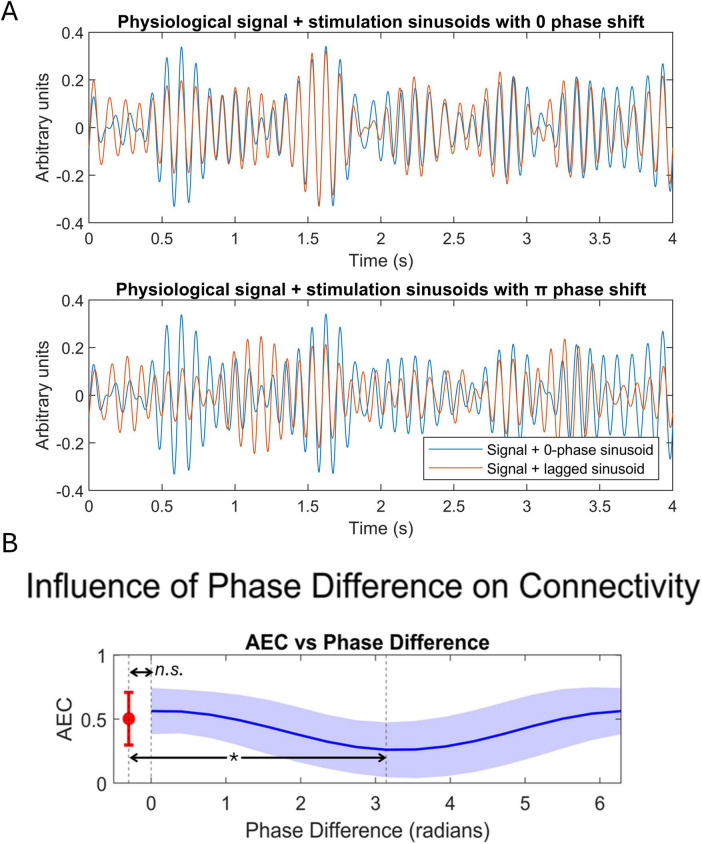
Results of the simulation of the effect of phase shifts tACS influence on sFC. **(A)** Physiological signals being stimulated with in-phase stimulation (top), and with anti-phase stimulation (bottom). **(B)** Relation between AEC and the phase difference between stimulation sinusoids. Significant reductions between anti-phase stimulation and initial connectivity were found and are indicated by an asterisk. Ns, non-significant.

We found that any phase shift between the stimuli deviating from 0 or 2π phase produced a decrease in AEC connectivity, as shown in Figure 7. Specifically, a significant difference in AEC values [t-stat = 7.3485, *p*-value < 0.0001] emerged when comparing the cases of no stimulation and anti-phase stimulation (phase = π). No significant differences were found when comparing connectivity in the case of no stimulation and stimulation with in-phase sinusoids EC [t-stat = −1.9688, *p*-value = 0.0805], even if it was close to statistical significance.

## 4 Discussion

In this study, we assessed the influence of a parieto-occipital IAF-tACS protocol on dynamic and static FC, measured by MEG, by comparing pre-stimulation, post-stimulation and ratio of both sessions of the *stim* and *sham* groups. The *stim* group showed a significant sFC reduction, spanning intra- and inter-DMN links, which affected the DMN-SN connectivity as well as the connectivity between posterior and antero-posterior hubs of the DMN. Importantly, the sFC ratio between DMN posterior regions also showed a negative correlation with their relative power ratio. Regarding dFC, the opposite pattern emerged, with an enhancement over sessions. The *stim* group showed a significant increase in dFC over intra- and inter-DMN links, although no statistically significant differences were found between the groups. Overall, these results complement previous literature by assessing the influence of personalized tACS on FC in healthy participants, as well as expand previous knowledge on the differences between sFC and dFC.

Our results reflect a widespread sFC reduction, spanning both intra- and inter-DMN connections under the triple network model. More specifically, both the connectivity among the nodes of the DMN and the connectivity between the DMN and the SN showed reductions in the *stim* group compared to the *sham* group. Additionally, the significant pre-post decreases of sFC in the *stim* group in those same links, with no significant changes in the *sham* group, strongly support that these changes were induced by the application of tACS. Interestingly, these results indicate that tACS had a stronger influence on the connectivity of the DMN than in the other networks of the model. This event is congruent with the stimulation protocol applied, which exerted maximum induced currents over the PCU and PCC that are important posterior hubs of the DMN. It also fits well with previous literature, which claim that IAF-tACS during resting state affects the ongoing alpha activity at the DMN (Cabrera-Álvarez et al., 2023; Clancy et al., 2022; Liu et al., 2018). At the same time, we find that the increased influence of tACS over the DMN-SN link compared to the DMN-CEN link is coherent with the triple network model, due to the increased activity correlation between the DMN and the SN than between DMN and CEN in both resting and task conditions (Menon, 2011).

Relevantly, the reductions in sFC between the PCU-ACC and PCU-PCC, revealed by the DMN connectivity *post hoc* analyses, are of special interest in the context of Alzheimer’s disease (AD). Specifically, an early rise in sFC between these areas is found in individuals at risk of disease development due to their family history (García-Colomo et al., 2024; Ramírez-Toraño et al., 2021). Moreover, the whole DMN is affected in the disease as its function, metabolic activity and structure become altered through the course of the disease (Badhwar et al., 2017). Some of these regions, such as the PCU, behave as hubs of the network and become vulnerable to the progression of the disease (Stam, 2014). In particular, these regions show increased excitability and connectivity in the earliest stages of Aβ accumulation, which intensifies the severity of the disease (López-Sanz et al., 2017; Maestú et al., 2021; Ranasinghe et al., 2022). Therefore, reducing or limiting the initial increase in neuronal excitability and connectivity through a non-invasive technique (such as tACS) could pose as a potential preventive treatment in prodromal stages of AD. Further investigation in this direction is needed, as well as in the cognitive effects of the proposed tACS protocol.

The results obtained in this study do not support our hypothesis of enhanced sFC after tACS, which was based on previous literature (Clancy et al., 2018; Schwab et al., 2019; Van Schouwenburg et al., 2017). However, a plausible explanation for our divergent results could stem from the lack of comparability in the field (Bikson et al., 2018), due to the use of different stimulation protocols, spatial targets, or means to measure the stimulation effects. Thus, for example, the study performed by Schwab et al. (2019) reported an increase in alpha functional static connectivity, calculated through imaginary coherence, for occipitoparietal in-phase tACS when compared to anti-phase and jittered-phase tACS. However, because a sham group was not included in their study our results do not stem from the same baseline conditions. Additionally, their tACS protocol was administered through HD-tACS, where the stimulation electrodes are surrounded by returning electrodes in an effort to focalize the elicited electric field. This is especially relevant, as the phase differences induced in the neural populations in the cortex would be highly dissimilar between protocols. This is the same case as in Clancy et al. (2018), which applied HD-tACS to occipito-parietal areas using the IAF as stimulation frequency, and additionally used Granger causality, a different sFC parameter than ours, to measure changes in connectivity. In the work by of Van Schouwenburg et al. (2017), both increases and decreases in FC were observed in different areas of the brain, and a second study by the same group could not replicate the results found in the first one (Van Schouwenburg et al., 2018). Finally, other studies based on fMRI-FC show inconclusive results, with some finding increases in sFC after tACS (Bächinger et al., 2017; Mondino et al., 2020) and other finding sFC reductions (Abellaneda-Pérez et al., 2020; Gundlach et al., 2020). Additionally, it is important to remark that some other conditions of our study might have played a role in our divergent results. These include a higher tACS intensity than in most previous work, which could be particularly relevant given possible non-linear effects of tACS intensity (Batsikadze et al., 2013), participants’ advanced age, or the specific method we used to address changes over FC by calculating FC ratios. In conclusion, there is a need for replication studies and a standardization of the stimulation protocols to evaluate the effects of tACS on sFC.

On the other hand, we hypothesize that the reductions in sFC found in this study might be explained by the interaction between the electric field direction and the position of pyramidal neurons along the folded cortex. Where the electric field direction is aligned parallel to the body axis of pyramidal cells, depolarization will be elicited. Inversely, where the alignment is antiparallel, hyperpolarization occurs. This introduces phase disparities in the stimulation induced and its intensity (Figure 6). To illustrate the possible effects of these phase disparities over sFC, we performed an ad-hoc analysis simulating the influence of tACS over physiological MEG signals. We found that any phase shift between the stimulatory sinusoids deviating from 0 or 2π phase produced a decrease in AEC connectivity, as shown in Figure 7. The phase difference between the stimulatory sinusoid waves modifies the envelopes differently, reaching a maximum misalignment of the original physiologic signals’ envelopes when the phase shift is that of π, consequently reducing their AEC. The interpretation of these simulation results should be considered with caution as many assumptions have been made. For instance, we have assumed a straightforward behavior and influence of tACS as sinusoidal signals over neuronal physiological signals, and considered white noise as a plausible proxy for neuronal activity. Nevertheless, we consider that this theoretical experiment adds to the understanding of the results obtained in this study.

For a better understanding of the electrophysiological phenomena underlying the use of tACS, we conducted an additional study on the joint behavior of sFC and power in the DMN links with significant changes after tACS. This revealed a consistent pattern of negative correlations between the change of sFC in the PCU-PCC link, and power change in these two regions for the *stim* group. Only the correlation with the PCU’s power ratio distinguished the behavior between the *sham* and *stim* groups, possibly explained by the fact that the precuneus is a hub region directly modulated by our stimulation protocol, or due to the reduced statistical power provided by our sample. Aligned with the previous explanation for the sFC reductions, tACS could introduce non-uniform phase distributions in the cortex, favoring a misalignment of envelopes, but still entraining the activity of the neuronal populations in the same stimulation frequency. This would consequently increase the activity within the IAF frequency range, increasing power (Cabrera-Álvarez et al., 2023; Zaehle et al., 2010) but reducing sFC as shown previously.

Finally, it is also important to comment on the different behavior observed when studying the effects of tACS on dFC. Our results reveal an increase in the intra-DMN dFC both in the *sham* and *stim* groups. However, the *stim* group also showed pre-post increases in inter-DMN links, following a similar trend to that observed in the sFC changes, but extending the alterations to the DMN-CEN link as well. This event might be explained by the fact that sFC dismisses relevant information throughout the averaging of connectivity features over time which, along with the opposite direction of dFC changes compared to sFC, supports the inclusion of dFC parameters in future electrophysiological studies.

Nevertheless, our experimental design did not allow us to ascertain whether there exists an effect of tACS on dFC or not, as no between-group differences were found. This might have happened due to the reduced sample size and the fact that dFC changes shared the same direction in both groups, and all we can do is consider the different possibilities behind these results. The absence of literature on tACS and dFC in this field did not facilitate the building of hypotheses, and hindered the interpretation of the study outcomes. The observed changes could stem from a common origin in both groups, such as the placebo-effect or tiredness. On the other hand, these results might reflect a differential effect of neuromodulation, and the observed increases of dFC in the *stim* could be explained by the induced decrease in sFC observed in this same group, while the increases of dFC in the *sham* group would originate from other sources, which is supported by the intragroup increases of dFC over more areas in the *stim* group than in the *sham* group. As a last possibility, we notice a common pattern of changes in dFC in both groups, with more pronounced increases in the links involving the DMN, then in the SN, and finally in the CEN, following the logic exposed in a previous paragraph. These increases are more pronounced in the *stim* group, reaching significance in additional areas, possibly reflecting a potentiation effect of tACS on the default effect of the experiment. Future studies should address this question, either by increasing the sample size, or through the application of techniques that allow for the study of dFC at a lower time-scale, such as EEG microstates or Hidden Markov Models (HMMs).

Some limitations of the present experiment must be noted. Firstly, while comparable to previous studies in the field, the sample size of our experiment was modest. This limited the possibility of introducing potential confounding variables as covariates. Nevertheless, no significant differences in any of the demographic variables between the *sham* and *stim* groups were observed. Future studies should consider larger sample sizes to increase statistical power. Importantly, even though physiological relative IAF ± 2 Hz power was not used as a covariate, since there were no significant differences in relative power between groups, the possibility for power to constitute a confounding factor in our study is slim. Secondly, the temporal length of the protocol could have induced fatigue in the participants, influencing the results in terms of power and FC (Ishii et al., 2013), and changing IAF between the *Pre* recording sessions and during the stimulation. Additionally, our tACS protocol was not phase-tuned to the brain oscillations, which could have had an impact on FC.

Our investigation lacks behavioral evaluation and consequently did not address the cognitive relevance of the findings. Also, the current literature presents elevated variability in the methods and stimulation used, yielding non-comparable results between studies. Therefore, future research should focus on standardizing methodologies, replicating previous results, including neuropsychological assessments, combining electrophysiological and fMRI measurements, as well as using personalized anatomical targeting to reduce inter-subject variability (Mikkonen et al., 2020; Miranda et al., 2018). The use of computational modeling to systematically simulate and study the differences between stimulation protocols would also contribute to knowledge convergence and experiment reproducibility in the field.

## 5 Conclusion

To conclude, this is, to the best of our knowledge, the first study assessing both dynamic and static FC changes induced by personalized IAF-tACS with MEG in healthy participants. Our results suggest that IAF-tACS over occipito-parietal areas reduced sFC, specifically over intra- and inter-DMN links. Based on our simulations, we hypothesize that this effect is mediated by the alternating in-phase and anti-phase neuromodulation that tACS produces in oppositely oriented cortices, which might lead to the reduction of amplitude-based synchrony. Future studies should address this possibility. Additionally, we find that the changes in sFC between posterior areas negatively correlated with power increases in the same frequency band. Lastly, our analyses revealed a significant pre-post increase in dFC over intra- and inter-DMN links in both groups, with no statistically significant between-group differences. Our findings highlight a potential treatment for functional connectivity reduction, potentially beneficial for AD, and expand the knowledge of the possible influence of brain folding on the individual effects of tACS.

## Data Availability

The datasets analyzed for this study, comprising clean MEG recordings, can be found in the following Dropbox link: The clean data used in this study can be found in the following dropbox folder: https://www.dropbox.com/sh/nap4v19b390ptxz/AAC8IvWFs5JpAF-4LzRpUE_Oa?dl=0. Further inquiries can directed to the corresponding author.

## References

[B1] Abellaneda-Pérez K. Vaqué-Alcázar L. Perellón-Alfonso R. Bargalló N. Kuo M.-F. Pascual-Leone A. (2020). Differential tDCS and tACS effects on working memory-related neural activity and resting-state connectivity. *Front. Neurosci.* 13:1440. 10.3389/fnins.2019.01440 32009896 PMC6978675

[B2] Aberra A. S. Wang B. Grill W. M. Peterchev A. V. (2020). Simulation of transcranial magnetic stimulation in head model with morphologically-realistic cortical neurons. *Brain Stimul.* 13 175–189. 10.1016/j.brs.2019.10.002 31611014 PMC6889021

[B3] Alves P. N. Foulon C. Karolis V. Bzdok D. Margulies D. S. Volle E. (2019). An improved neuroanatomical model of the default-mode network reconciles previous neuroimaging and neuropathological findings. *Commun. Biol.* 2:370. 10.1038/s42003-019-0611-3 31633061 PMC6787009

[B4] Antal A. Paulus W. (2013). Transcranial alternating current stimulation (tACS). *Front. Hum. Neurosci.* 7:317. 10.3389/fnhum.2013.00317 23825454 PMC3695369

[B5] Antal A. Boros K. Poreisz C. Chaieb L. Terney D. Paulus W. (2008). Comparatively weak after-effects of transcranial alternating current stimulation (tACS) on cortical excitability in humans. *Brain Stimul.* 1 97–105. 10.1016/j.brs.2007.10.001 20633376

[B6] Babiloni C. Frisoni G. Pievani M. Vecchio F. Lizio R. Buttiglione M. (2009). Hippocampal volume and cortical sources of EEG alpha rhythms in mild cognitive impairment and Alzheimer disease. *NeuroImage* 44 123–135. 10.1016/j.neuroimage.2008.08.005 18805495

[B7] Bächinger M. Zerbi V. Moisa M. Polania R. Liu Q. Mantini D. (2017). Concurrent tACS-fMRI reveals causal influence of power synchronized neural activity on resting state fMRI connectivity. *J. Neurosci.* 37 4766–4777. 10.1523/JNEUROSCI.1756-16.2017 28385876 PMC6596494

[B8] Badhwar A. Tam A. Dansereau C. Orban P. Hoffstaedter F. Bellec P. (2017). Resting-state network dysfunction in Alzheimer’s disease: A systematic review and meta-analysis. *Alzheimer’s Dement. Diagnosis Assess. Dis. Monit.* 8 73–85. 10.1016/j.dadm.2017.03.007 28560308 PMC5436069

[B9] Batsikadze G. Moliadze V. Paulus W. Kuo M. F. Nitsche M. A. (2013). Partially non-linear stimulation intensity-dependent effects of direct current stimulation on motor cortex excitability in humans. *J. Physiol.* 591 1987–2000. 10.1113/jphysiol.2012.249730 23339180 PMC3624864

[B10] Belouchrani A. Abed-Meraim K. Cardoso J.-F. Moulines E. (1997). A blind source separation technique using second-order statistics. *IEEE Trans. Signal Process.* 45 434–444. 10.1109/78.554307

[B11] Benjamini Y. Hochberg Y. (1995). Controlling the false discovery rate: A practical and powerful approach to multiple testing. *J. R. Stat. Soc. Ser. B (Methodological)* 57 289–300. 10.1111/j.2517-6161.1995.tb02031.x

[B12] Bikson M. Brunoni A. R. Charvet L. E. Clark V. P. Cohen L. G. Deng Z.-D. (2018). Rigor and reproducibility in research with transcranial electrical stimulation: An NIMH-sponsored workshop. *Brain Stimul.* 11 465–480. 10.1016/j.brs.2017.12.008 29398575 PMC5997279

[B13] Brunoni A. R. Amadera J. Berbel B. Volz M. S. Rizzerio B. G. Fregni F. (2011). A systematic review on reporting and assessment of adverse effects associated with transcranial direct current stimulation. *Int. J. Neuropsychopharmacol.* 14 1133–1145. 10.1017/S1461145710001690 21320389

[B14] Buzsáki G. (2006). *Rhythms of the Brain.* Oxford: Oxford University Press, 10.1093/acprof:oso/9780195301069.001.0001

[B15] Cabrera-Álvarez J. Sánchez-Claros J. Carrasco-Gómez M. Del Cerro-León A. Gómez-Ariza C. J. Maestú F. (2023). Understanding the effects of cortical gyrification in tACS: Insights from experiments and computational models. *Front. Neurosci.* 17:1223950. 10.3389/fnins.2023.1223950 37655010 PMC10467425

[B16] Canuet L. Pusil S. Lopez M. E. Bajo R. Pineda-Pardo J. A. Cuesta P. (2015). Network disruption and cerebrospinal fluid amyloid-beta and phospho-tau levels in mild cognitive impairment. *J. Neurosci.* 35 10325–10330. 10.1523/JNEUROSCI.0704-15.2015 26180207 PMC6605340

[B17] Carrasco-Gómez M. García-Colomo A. Nebreda A. Bruña R. Santos A. Maestú F. (2024). Dynamic functional connectivity is modulated by the amount of p-Tau231 in blood in cognitively intact participants. *bioRxiv [Preprint]* 10.1101/2024.05.29.596323 38854147 PMC11160744

[B18] Chaieb L. Antal A. Paulus W. (2011). Transcranial alternating current stimulation in the low kHz range increases motor cortex excitability. *Restorative Neurol. Neurosci.* 29 167–175. 10.3233/RNN-2011-0589 21586823

[B19] Clancy K. J. Andrzejewski J. A. You Y. Rosenberg J. T. Ding M. Li W. (2022). Transcranial stimulation of alpha oscillations up-regulates the default mode network. *Proc. Natl. Acad. Sci.* 119:e2110868119. 10.1073/pnas.2110868119 34969856 PMC8740757

[B20] Clancy K. J. Baisley S. K. Albizu A. Kartvelishvili N. Ding M. Li W. (2018). Lasting connectivity increase and anxiety reduction via transcranial alternating current stimulation. *Soc. Cogn. Affect. Neurosci.* 13 1305–1316. 10.1093/scan/nsy096 30380131 PMC6277743

[B21] Colclough G. L. Woolrich M. W. Tewarie P. K. Brookes M. J. Quinn A. J. Smith S. M. (2016). How reliable are MEG resting-state connectivity metrics? *NeuroImage* 138 284–293. 10.1016/j.neuroimage.2016.05.070 27262239 PMC5056955

[B22] De Koninck B. P. Brazeau D. Guay S. Herrero Babiloni A. De Beaumont L. (2023). Transcranial alternating current stimulation to modulate alpha activity: A systematic review. *Neuromodulation Technol. Neural Interface* 26 1549–1584. 10.1016/j.neurom.2022.12.007 36725385

[B23] Elyamany O. Leicht G. Herrmann C. S. Mulert C. (2021). Transcranial alternating current stimulation (tACS): From basic mechanisms towards first applications in psychiatry. *Eur. Arch. Psychiatry Clin. Neurosci.* 271 135–156. 10.1007/s00406-020-01209-9 33211157 PMC7867505

[B24] Friston K. J. (1994). Functional and effective connectivity in neuroimaging: A synthesis. *Hum. Brain Mapp.* 2 56–78. 10.1002/hbm.460020107

[B25] Friston K. J. (2011). Functional and effective connectivity: A review. *Brain Connectivity* 1 13–36. 10.1089/brain.2011.0008 22432952

[B26] Garcés P. López-Sanz D. Maestú F. Pereda E. (2017). Choice of magnetometers and gradiometers after signal space separation. *Sensors* 17:2926. 10.3390/s17122926 29258189 PMC5751446

[B27] García-Colomo A. Nebreda A. Carrasco-Gómez M. de Frutos-Lucas J. Ramirez-Toraño F. Spuch C. (2024). Longitudinal changes in the functional connectivity of individuals at risk of Alzheimer’s disease. *GeroScience* 46 2989–3003. 10.1007/s11357-023-01036-5 38172488 PMC11009204

[B28] Gundlach C. Müller M. M. Hoff M. Ragert P. Nierhaus T. Villringer A. (2020). Reduction of somatosensory functional connectivity by transcranial alternating current stimulation at endogenous mu-frequency. *NeuroImage* 221:117175. 10.1016/j.neuroimage.2020.117175 32682989

[B29] Hämäläinen M. Hari R. Ilmoniemi R. J. Knuutila J. Lounasmaa O. V. (1993). Magnetoencephalography—Theory, instrumentation, and applications to noninvasive studies of the working human brain. *Rev. Modern Phys.* 65 413–497. 10.1103/RevModPhys.65.413

[B30] Helfrich R. F. Knepper H. Nolte G. Strüber D. Rach S. Herrmann C. S. (2014). Selective modulation of interhemispheric functional connectivity by HD-tACS shapes perception. *PLoS Biol.* 12:e1002031. 10.1371/journal.pbio.1002031 25549264 PMC4280108

[B31] Hinkle D. E. Wiersma W. Jurs S. G. (1988). *Applied Statistics for the Behavioral Sciences.* Jurs Boston: Houghton Mifflin Co.

[B32] Huang Y. Datta A. Bikson M. Parra L. C. (2019). Realistic volumetric-approach to simulate transcranial electric stimulation—ROAST—a fully automated open-source pipeline. *J. Neural Eng.* 16:056006. 10.1088/1741-2552/ab208d 31071686 PMC7328433

[B33] Ishii A. Tanaka M. Shigihara Y. Kanai E. Funakura M. Watanabe Y. (2013). Neural effects of prolonged mental fatigue: A magnetoencephalography study. *Brain Res.* 1529 105–112. 10.1016/j.brainres.2013.07.022 23880373

[B34] Jensen O. Mazaheri A. (2010). Shaping functional architecture by oscillatory alpha activity: Gating by inhibition. *Front. Hum. Neurosci.* 4:186. 10.3389/fnhum.2010.00186 21119777 PMC2990626

[B35] Kar K. Ito T. Cole M. W. Krekelberg B. (2020). Transcranial alternating current stimulation attenuates BOLD adaptation and increases functional connectivity. *J. Neurophysiol.* 123 428–438. 10.1152/jn.00376.2019 31825706 PMC6985864

[B36] Kasten F. H. Dowsett J. Herrmann C. S. (2016). Sustained aftereffect of α-tACS lasts Up to 70 min after stimulation. *Front. Hum. Neurosci.* 10:245. 10.3389/fnhum.2016.00245 27252642 PMC4879138

[B37] Knuth K. H. (2019). Optimal data-based binning for histograms and histogram-based probability density models. *Digital Signal Process.* 95:102581. 10.1016/j.dsp.2019.102581

[B38] Liu A. Vöröslakos M. Kronberg G. Henin S. Krause M. R. Huang Y. (2018). Immediate neurophysiological effects of transcranial electrical stimulation. *Nat. Commun.* 9:5092. 10.1038/s41467-018-07233-7 30504921 PMC6269428

[B39] López-Sanz D. Bruña R. Garcés P. Camara C. Serrano N. Rodríguez-Rojo I. C. (2016). Alpha band disruption in the AD-continuum starts in the subjective cognitive decline stage: A MEG study. *Sci. Rep.* 6:37685. 10.1038/srep37685 27883082 PMC5121589

[B40] López-Sanz D. Bruña R. Garcés P. Martín-Buro M. C. Walter S. Delgado M. L. (2017). Functional connectivity disruption in subjective cognitive decline and mild cognitive impairment: A common pattern of alterations. *Front. Aging Neurosci.* 9:109. 10.3389/fnagi.2017.00109 28484387 PMC5399035

[B41] Maestú F. de Haan W. Busche M. A. DeFelipe J. (2021). Neuronal excitation/inhibition imbalance: Core element of a translational perspective on Alzheimer pathophysiology. *Ageing Res. Rev.* 69:101372. 10.1016/j.arr.2021.101372 34029743

[B42] Menon V. (2011). Large-scale brain networks and psychopathology: A unifying triple network model. *Trends Cogn. Sci.* 15 483–506. 10.1016/j.tics.2011.08.003 21908230

[B43] Mikkonen M. Laakso I. Tanaka S. Hirata A. (2020). Cost of focality in TDCS: Interindividual variability in electric fields. *Brain Stimul.* 13 117–124. 10.1016/j.brs.2019.09.017 31606449

[B44] Miranda P. C. Callejón-Leblic M. A. Salvador R. Ruffini G. (2018). Realistic modeling of transcranial current stimulation: The electric field in the brain. *Curr. Opin. Biomed. Eng.* 8 20–27. 10.1016/j.cobme.2018.09.002

[B45] Mondino M. Ghumman S. Gane C. Renauld E. Whittingstall K. Fecteau S. (2020). Effects of transcranial stimulation with direct and alternating current on resting-state functional connectivity: An exploratory study simultaneously combining stimulation and multiband functional magnetic resonance imaging. *Front. Hum. Neurosci.* 13:474. 10.3389/fnhum.2019.00474 32116597 PMC7012783

[B46] O’Neill G. C. Barratt E. L. Hunt B. A. E. Tewarie P. K. Brookes M. J. (2015). Measuring electrophysiological connectivity by power envelope correlation: A technical review on MEG methods. *Phys. Med. Biol.* 60 R271–R295. 10.1088/0031-9155/60/21/R271 26447925

[B47] Oostenveld R. Fries P. Maris E. Schoffelen J.-M. (2011). FieldTrip: Open source software for advanced analysis of MEG, EEG, and invasive electrophysiological data. *Comput. Intell. Neurosci.* 2011 1–9. 10.1155/2011/156869 21253357 PMC3021840

[B48] Polanía R. Nitsche M. A. Korman C. Batsikadze G. Paulus W. (2012). The importance of timing in segregated theta phase-coupling for cognitive performance. *Curr. Biol.* 22 1314–1318. 10.1016/j.cub.2012.05.021 22683259

[B49] Preisig B. C. Riecke L. Sjerps M. J. Kösem A. Kop B. R. Bramson B. (2021). Selective modulation of interhemispheric connectivity by transcranial alternating current stimulation influences binaural integration. *Proc. Natl. Acad. Sci.* 118:e2015488118. 10.1073/pnas.2015488118 33568530 PMC7896308

[B50] Raichle M. E. (2011). The restless brain. *Brain Connectivity* 1 3–12. 10.1089/brain.2011.0019 22432951 PMC3621343

[B51] Raichle M. E. (2015). The brain’s default mode network. *Annu. Rev. Neurosci.* 38 433–447. 10.1146/annurev-neuro-071013-014030 25938726

[B52] Ramírez-Toraño F. Bruña R. de Frutos-Lucas J. Rodríguez-Rojo I. C. Marcos, de Pedro S. (2021). Functional connectivity hypersynchronization in relatives of Alzheimer’s disease patients: An early E/I balance dysfunction? *Cereb. Cortex* 31 1201–1210. 10.1093/cercor/bhaa286 33108468

[B53] Ranasinghe K. G. Kudo K. Hinkley L. Beagle A. Lerner H. Mizuiri D. (2022). Neuronal synchrony abnormalities associated with subclinical epileptiform activity in early-onset Alzheimer’s disease. *Brain* 145 744–753. 10.1093/brain/awab442 34919638 PMC9630715

[B54] Schwab B. C. Misselhorn J. Engel A. K. (2019). Modulation of large-scale cortical coupling by transcranial alternating current stimulation. *Brain Stimul.* 12 1187–1196. 10.1016/j.brs.2019.04.013 31101568

[B55] Shannon C. E. (1948). A mathematical theory of communication. *Bell Syst. Techn. J.* 27 379–423. 10.1002/j.1538-7305.1948.tb01338.x

[B56] Stam C. J. (2014). Modern network science of neurological disorders. *Nat. Rev. Neurosci.* 15 683–695. 10.1038/nrn3801 25186238

[B57] Tan J. Iyer K. K. Tang A. D. Jamil A. Martins R. N. Sohrabi H. R. (2019). Modulating functional connectivity with non-invasive brain stimulation for the investigation and alleviation of age-associated declines in response inhibition: A narrative review. *NeuroImage* 185 490–512. 10.1016/j.neuroimage.2018.10.044 30342977

[B58] Taulu S. Simola J. (2006). Spatiotemporal signal space separation method for rejecting nearby interference in MEG measurements. *Phys. Med. Biol.* 51 1759–1768. 10.1088/0031-9155/51/7/008 16552102

[B59] Tzourio-Mazoyer N. Landeau B. Papathanassiou D. Crivello F. Etard O. Delcroix N. (2002). Automated anatomical labeling of activations in SPM using a macroscopic anatomical parcellation of the MNI MRI single-subject brain. *NeuroImage* 15 273–289. 10.1006/nimg.2001.0978 11771995

[B60] Utevsky A. V. Smith D. V. Huettel S. A. (2014). Precuneus is a functional core of the default-mode network. *J. Neurosci.* 34 932–940. 10.1523/JNEUROSCI.4227-13.2014 24431451 PMC3891968

[B61] Van Schouwenburg M. R. Sörensen L. K. A. De Klerk R. Reteig L. C. Slagter H. A. (2018). No differential effects of two different alpha-band electrical stimulation protocols over fronto-parietal regions on spatial attention. *Front. Neurosci.* 12:433. 10.3389/fnins.2018.00433 30018530 PMC6037819

[B62] Van Schouwenburg M. R. Zanto T. P. Gazzaley A. (2017). Spatial attention and the effects of frontoparietal alpha band stimulation. *Front. Hum. Neurosci.* 10:658. 10.3389/fnhum.2016.00658 28174529 PMC5259681

[B63] Van Veen B. D. Van Drongelen W. Yuchtman M. Suzuki A. (1997). Localization of brain electrical activity via linearly constrained minimum variance spatial filtering. *IEEE Trans. Biomed. Eng.* 44 867–880. 10.1109/10.623056 9282479

[B64] Vogeti S. Boetzel C. Herrmann C. S. (2022). Entrainment and spike-timing dependent plasticity – A review of proposed mechanisms of transcranial alternating current stimulation. *Front. Syst. Neurosci.* 16:827353. 10.3389/fnsys.2022.827353 35283735 PMC8909135

[B65] Vossen A. Gross J. Thut G. (2015). Alpha power increase after transcranial alternating current stimulation at alpha frequency (α-tACS) reflects plastic changes rather than entrainment. *Brain Stimul.* 8 499–508. 10.1016/j.brs.2014.12.004 25648377 PMC4464304

[B66] Wang Y. Hou P. Li W. Zhang M. Zheng H. Chen X. (2022). The influence of different current-intensity transcranial alternating current stimulation on the eyes-open and eyes-closed resting-state electroencephalography. *Front. Hum. Neurosci.* 16:934382. 10.3389/fnhum.2022.934382 36061496 PMC9429605

[B67] Zaehle T. Rach S. Herrmann C. S. (2010). Transcranial alternating current stimulation enhances individual alpha activity in human EEG. *PLoS One* 5:e13766. 10.1371/journal.pone.0013766 21072168 PMC2967471

[B68] Zarubin G. Gundlach C. Nikulin V. Villringer A. Bogdan M. (2020). Transient amplitude modulation of alpha-band oscillations by short-time intermittent closed-loop tACS. *Front. Hum. Neurosci.* 14:366. 10.3389/fnhum.2020.00366 33100993 PMC7500443

